# *Salmonella* Typhimurium infection complicating very early-onset inflammatory bowel disease presenting with Pseudo-intussusception: a case report

**DOI:** 10.3389/fped.2026.1885979

**Published:** 2026-08-18

**Authors:** Qian Liu, Ling Yang, Xuefeng Sun, Yishan Liu, Hui Yang

**Affiliations:** 1 The Second People’s Hospital of China Three Gorges University, The Second People’s Hospital of Yichang, Yichang, China; 2 College of Medicine and Health Sciences, China Three Gorges University, Yichang, China

**Keywords:** case report, elevated serum igE, hematochezia, pseudo-intussusception, *Salmonella* Typhimurium, very early-onset inflammatory bowel disease

## Abstract

The clinical manifestations of acute non-typhoidal *Salmonella* infection can overlap with underlying very early-onset inflammatory bowel disease (VEO-IBD), complicating early differential diagnosis. We report a 5-year-old boy who presented with fever, paroxysmal abdominal pain, and high-volume watery hematochezia. Initial abdominal ultrasonography revealed a “concentric ring sign” mimicking intussusception, while laboratory tests concurrently demonstrated elevated serum total immunoglobulin E (IgE) levels. Subsequent water-soluble gastrointestinal contrast studies and computed tomography ruled out mechanical intestinal obstruction. Early endoscopy and mucosal biopsy revealed cryptitis and chronic active inflammation from the rectum to the sigmoid colon, and stool cultures isolated *Salmonella* Typhimurium, with serum total IgE peaking at 2965 IU/mL. Based on these clinical findings, the patient was diagnosed with concurrent acute *Salmonella* Typhimurium infection and VEO-IBD. The patient received a combined regimen of systemic intravenous cefotaxime sodium and localized therapy comprising dexamethasone retention enemas and mesalazine suppositories, with symptom resolution within 10 days. This case illustrates the value of multimodal imaging and early endoscopy in differentiating overlapping enteric infections from VEO-IBD, and suggests that integrating systemic antimicrobials with localized anti-inflammatory agents may be a feasible therapeutic strategy.

## 1 Introduction

Recent epidemiological data indicate that pediatric-onset inflammatory bowel disease (IBD) incidence continues to rise in most reporting regions worldwide; however, data on very early-onset inflammatory bowel disease (VEO-IBD) remain limited to countries with historically high rates, exhibiting geographic variations and heterogeneous trends over time (1). Ulcerative colitis (UC), a major subtype of IBD, presents unique challenges in this demographic. Pediatric UC features extensive and severe lesions, common atypical phenotypes, high relapse rates, and a risk of disease progression (2). Although the exact pathogenesis of UC remains unclear, several associated factors have been identified. These include dysregulated immune responses, gut microbiota alterations, genetic susceptibility, and environmental factors (3). Among these, enteric pathogen infections, such as *Salmonella* spp., can disrupt the intestinal epithelial barrier and may act as environmental triggers that incite or exacerbate IBD in genetically susceptible individuals.

Concurrent acute *Salmonella* Typhimurium infection and VEO-IBD is rare in children, and their overlapping acute symptoms and atypical imaging findings complicate early diagnosis. On sonographic evaluation, intestinal inflammation and submucosal edema may present as a “target sign” or “concentric ring sign,” which can mimic mechanical intussusception. Without a palpable mass or peritoneal signs, these sonographic findings confound early differentiation. Serum total immunoglobulin E (IgE) levels exceeding 2000 IU/mL are atypical in pediatric UC and may prompt the evaluation for primary immunodeficiency disorders. In children with an atopic predisposition, this marker may reflect a secondary T helper type 2 (Th2)-polarized immune response following intestinal mucosal barrier disruption.

This report describes a 5-year-old boy admitted to the hospital with fever and lower gastrointestinal bleeding, accompanied by ultrasound findings suggestive of intussusception. He also presented with significantly elevated serum total IgE levels and was diagnosed with *Salmonella* Typhimurium infection complicated by VEO-IBD. The aim of this report is to discuss the differential diagnosis and medical management strategies for cases involving these overlapping pathological conditions.

## 2 Case description

### 2.1 Patient information and clinical findings

**Figure 1:**
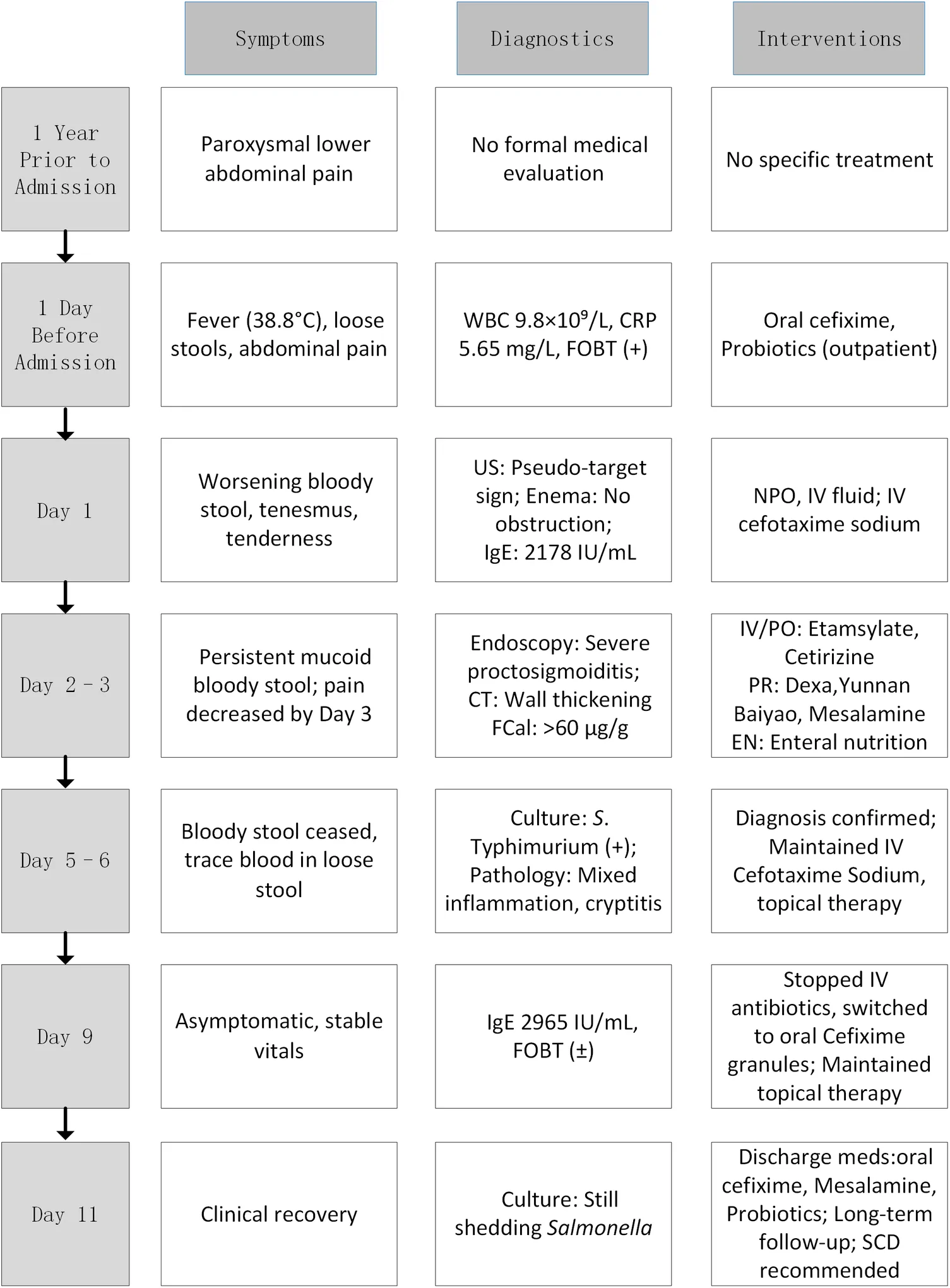
Clinical Timeline of the Patient's Episode of Care. The flowchart delineates the chronological progression of the patient's clinical symptoms, diagnostic evaluations, and therapeutic interventions from 1 year prior to admission through hospital discharge. CRP, C-reactive protein; EN, enteral nutrition; FCal, fecal calprotectin; FOBT, fecal occult blood test; IV, intravenous; NPO, nil per os; PR, per rectum; SCD, specific carbohydrate diet; US, ultrasound; WBC, white blood cell.

The chronological progression of the patient's clinical symptoms, diagnostic evaluations, and therapeutic interventions is summarized in Figure 1. A 5-year-old boy presented to the emergency department with a one-day history of irregular fever, intermittent abdominal pain, and diarrhea, which had rapidly progressed to high-volume watery hematochezia over the preceding 12 h. One day prior to admission, the patient developed a fever peaking at 38.8 °C, accompanied by lower-mid abdominal pain and the passage of yellow, loose stools on six occasions. By the early morning of admission, the patient experienced three consecutive episodes of watery hematochezia. The patient had a history of recurrent abdominal pain over the past year (with frequent exacerbations over the preceding 6 months). Outpatient examinations three months prior to this admission revealed weakly positive fecal occult blood and enlarged mesenteric lymph nodes on abdominal ultrasonography. He had a history of allergies to pollen and dust, which manifested as mild rashes. Family history was negative for primary immunodeficiencies and inflammatory bowel disease.

Upon admission, the patient's vital signs were as follows: temperature, 36.8 °C; heart rate, 92 beats/min; and respiratory rate, 21 breaths/min. Abdominal examination revealed a flat and soft abdomen with localized lower-mid abdominal tenderness, normoactive bowel sounds, and an absence of rebound tenderness, guarding, or palpable masses. Initial laboratory investigations revealed a white blood cell count of 9.8 × 10^9^/L (neutrophils 75.3%) and a C-reactive protein (CRP) level of 5.65 mg/L.

### 2.2 Diagnostic assessment

#### 2.2.1 Imaging studies

**Figure 2:**
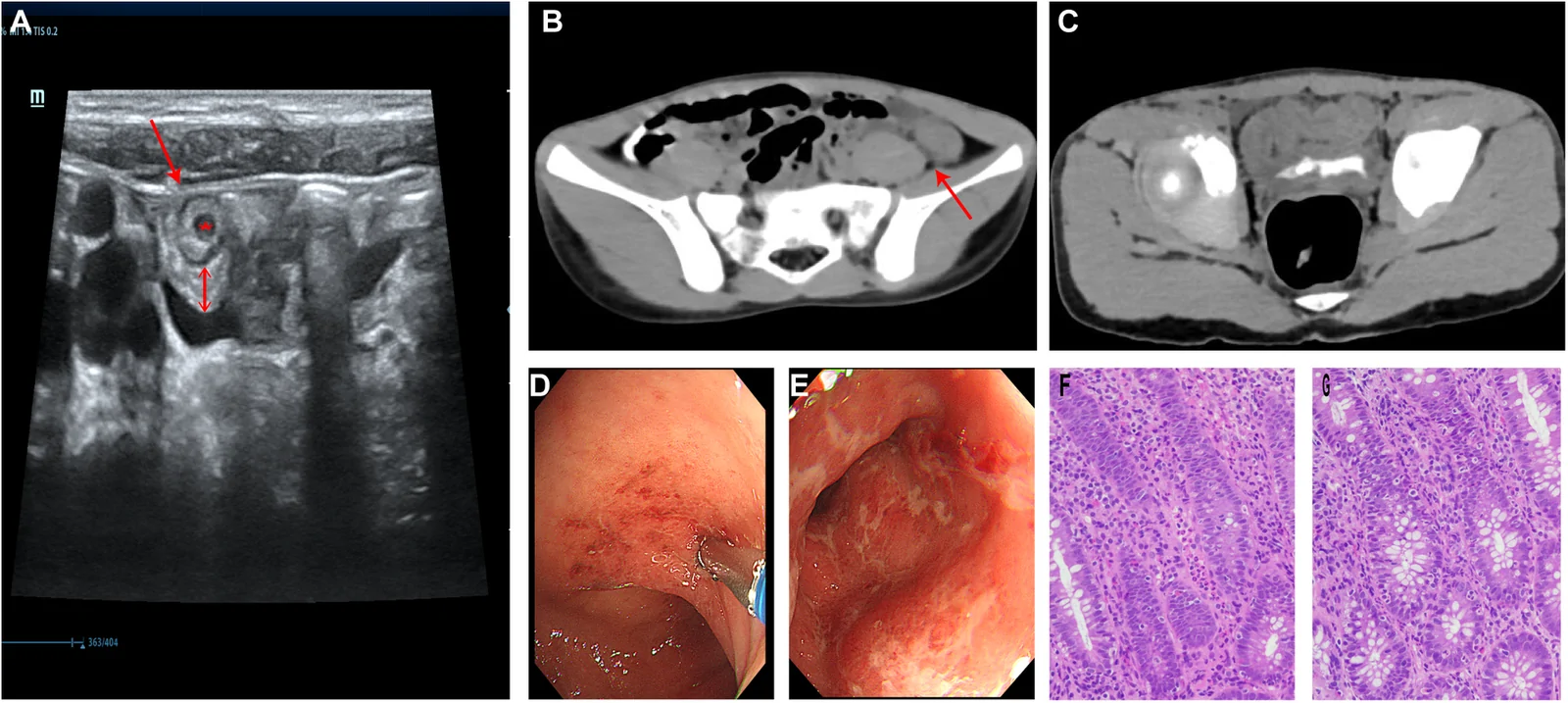
Comprehensive Composite of Radiological, Endoscopic, and Histopathological Findings. **(A)** Abdominal ultrasonography performed on the first day of admission demonstrating a pseudo “concentric ring sign” or “target sign” (indicated by the red arrow), initially mimicking small bowel intussusception. The asterisk indicates the central mucosal hyperechogenicity, and the double-headed arrow delineates the edematous bowel wall thickening. **(B,C)** Unenhanced abdominal computed tomography (CT) revealing continuous thickening of the sigmoid colon wall (indicated by the red arrow in B) accompanied by perienteric fluid accumulation, with no evidence of mechanical obstruction. **(D,E)** Colonoscopic view showing mucosal inflammation characterized by hyperemia, edema, loss of normal vascular pattern, and scattered superficial erosions in the rectum and sigmoid colon. **(F,G)** Hematoxylin and eosin (H&E) staining of colonic mucosal biopsies exhibiting disrupted crypt architecture, cryptitis, crypt abscess formation, and a mixed inflammatory cell infiltrate (predominantly plasma cells and neutrophils) extending throughout the lamina propria.

On the first day of admission, an abdominal ultrasound revealed a “concentric ring sign” suggestive of a possible small bowel intussusception (Figure 2A). Specifically, this initial sonographic evaluation demonstrated pronounced, symmetrical bowel wall thickening with preserved mural stratification and diffuse mucosal hyperechogenicity. Color Doppler imaging showed marked hypervascularity within the thickened submucosa, alongside the absence of trapped mesenteric fat or lymph nodes within the lesion. Subsequently, a water-soluble gastrointestinal contrast study was performed. The following day, an unenhanced abdominal CT scan revealed sigmoid colon wall thickening accompanied by minimal perienteric fluid (Figures 2B,C). Multimodal imaging confirmed the absence of mechanical obstruction.

#### 2.2.2 Endoscopy and pathology

Colonoscopy revealed diffuse hyperemia, edema, erosions, and ulcerative inflammatory changes in the mucosa from the upper rectum to the sigmoid colon (Figures 2D,E). According to the Paris classification for pediatric inflammatory bowel disease, the disease extent was classified as E2 (left-sided colitis) (4). The initial endoscopic assessment was limited to the rectosigmoid region; evaluation of the upper gastrointestinal tract and terminal ileum was deferred pending the resolution of the acute infectious episode. Microscopic evaluation of hematoxylin and eosin (H&E)-stained mucosal biopsy specimens demonstrated disrupted crypt architecture, florid cryptitis, and an abundant mixed inflammatory cell infiltrate—predominantly plasma cells and neutrophils—extending throughout the lamina propria (Figures 2F,G). Plasma cell infiltration was observed in the basal layer. Based on the comprehensive assessment of the clinical history, endoscopic evaluations, and histopathological findings, a diagnosis of VEO-IBD was established. Laboratory and pathogen testing: On the first day of hospitalization, a rapid semi-quantitative assay for fecal calprotectin was positive (>60 *μ*g/g). Concurrently, total serum IgE was measured at 2178 IU/mL; by the ninth day of hospitalization, it had further increased to 2965 IU/mL (reference range <100 IU/mL). Consecutive fecal occult blood tests were strongly positive. Furthermore, independent stool cultures obtained on hospital days 5 and 11 both yielded *Salmonella* Typhimurium.

### 2.3 Therapeutic intervention

The patient received systemic antimicrobial therapy combined with local anti-inflammatory treatment:

#### 2.3.1 Systemic antimicrobial therapy

Intravenous cefotaxime sodium (0.85 g, twice daily) was initiated. This regimen was continued after antimicrobial susceptibility testing confirmed sensitivity to third-generation cephalosporins.

#### 2.3.2 Localized anti-inflammatory and hemostatic therapy

On hospital day 3, a localized therapeutic regimen was initiated. A retention enema comprising dexamethasone sodium phosphate (2.5 mg) and Yunnan Baiyao powder (250 mg) was administered daily. This was complemented by the rectal administration of mesalazine suppositories (0.5 g). Concurrently, intravenous etamsylate was administered.

#### 2.3.3 Supportive care and microbiome modulation

During the acute phase, the patient was placed on a nil per os (NPO) regimen supported by intravenous fluid resuscitation. Upon symptom stabilization, an oral multi-strain probiotic formulation (containing live *Bifidobacterium*, *Lactobacillu*s, *Enterococcus*, and *Bacillus*) was administered. Additionally, oral cetirizine was administered. Following the complete resolution of abdominal pain, peptide-based enteral nutrition was progressively introduced.

### 2.4 Follow-up and outcomes

The patient tolerated all therapeutic interventions well, with no drug-related adverse reactions observed throughout the clinical course. By the fifth day of hospitalization, the gross bloody stools had ceased. By the tenth day, the abdominal pain had resolved, the stools had turned yellow and paste-like, and a repeat fecal occult blood test was negative. No further episodes of fever occurred during hospitalization, and vital signs remained stable. On the 11th day of hospitalization, the stool culture remained positive for *Salmonella*; the patient was afebrile, free of abdominal pain, hematochezia, and vomiting, with normal bowel sounds, and was discharged. Discharge instructions included oral cefixime granules (50 mg twice daily) for one week. The family was instructed to implement household infection-control measures, including hand hygiene and the disinfection of sanitary facilities, until fecal clearance is confirmed. The post-discharge follow-up plan included repeating the fecal occult blood test after 3 days, repeating the stool culture after 1 week, and a follow-up colonoscopy scheduled at 3 months. The patient was also advised to continue using mesalazine suppositories and live *Bifidobacterium* tablets, and to follow a specific carbohydrate diet (SCD). The medical team has established a long-term outpatient follow-up plan for the patient. Two outpatient follow-up visits at 1 week and 2 months after discharge revealed no severe symptom recurrence or hospital readmission, and growth parameters were within normal limits. Stool microscopy at both visits demonstrated the absence of red and white blood cells, with fecal occult blood turning negative at the 2-month follow-up.

## 3 Discussion

This report details a rare pediatric case characterized by a dual pathological overlap. An acute *Salmonella* Typhimurium infection was concurrent with severe lower gastrointestinal bleeding, subsequently leading to a diagnosis of VEO-IBD. The clinical course of this case provides valuable insights into imaging differentiation, overlapping pathophysiological mechanisms, and the selection of treatment strategies.

First, this case demonstrates how inflammatory pseudo-obstruction can confound the differential diagnosis of the acute abdomen. The patient's initial abdominal ultrasound revealed a “concentric ring sign” suggestive of intussusception. This imaging artifact is typically caused by diffuse submucosal congestion, intestinal wall edema, and localized smooth muscle spasms during the acute phase of severe inflammation. Initial sonographic findings indicated small intestinal thickening, whereas subsequent unenhanced CT and colonoscopic histopathology confirmed that the core inflammatory epicenter was localized to the rectum and sigmoid colon. This anatomical discrepancy suggests that severe enteritis-related spasm or local inflammation may compromise ultrasound accuracy. Given the absence of typical signs of peritoneal irritation or a palpable mass, a multimodal assessment was performed to clarify the diagnosis. The water-soluble gastrointestinal contrast study demonstrated slightly delayed transit but no evidence of complete mechanical obstruction. Furthermore, the subsequent unenhanced CT scan confirmed severe inflammatory wall thickening and perienteric fluid in the sigmoid colon, without showing any bowel-within-bowel invagination. This multimodal approach effectively differentiated severe inflammatory wall edema from true mechanical intussusception, thereby avoiding unnecessary surgical exploration and allowing the safe initiation of conservative medical therapy. This strategy thus provided a safe window for subsequent medical intervention. Contemporary pediatric inflammatory bowel disease guidelines highlight that colonic ultrasound, when available, can be utilized alongside fecal calprotectin to monitor disease activity, though data on its use remain limited (5). Severe autoimmune intestinal inflammation can present as an acute abdominal condition or pseudo-obstruction. Yang et al. (6) reported the case of a 13-year-old female patient with ulcerative colitis who experienced recurrent pseudo-obstruction associated with proteinase 3 antineutrophil cytoplasmic antibody (PR3-ANCA) positivity. This further underscores the need for multidimensional, in-depth differential diagnosis of atypical imaging phenotypes.

Second, regarding pathophysiology, a potential synergistic relationship exists between bacterial infections and latent VEO-IBD. The child's history of paroxysmal abdominal pain suggested that the intestinal mucosa was already in a state of chronic, low-grade inflammation. This environment likely reduced local colonization resistance, thereby increasing susceptibility to pathogenic microorganisms. *Salmonella* infection can alter the permeability of the intestinal epithelial barrier, induce the release of pro-inflammatory factors, and alter the composition of the gut microbiota; in genetically susceptible individuals, these changes can trigger the clinical onset or exacerbate an underlying state of inflammatory bowel disease (7). However, causality cannot be definitively established from a single case; alternative explanations, including severe infectious colitis mimicking IBD or an acute exacerbation of pre-existing latent VEO-IBD, must also be considered. Phenotypically, such superimposed enteric infections frequently mirror the initial onset or an acute severe flare of underlying IBD, substantially amplifying the complexity of early clinical differentiation. Notably, the patient exhibited only a minimally elevated C-reactive protein (CRP) level despite severe local colonic inflammation and bloody diarrhea, suggesting that initial systemic inflammatory markers may not always fully reflect the extent of mucosal injury in such overlapping conditions. As summarized in Table 1, previous reports on the overlap between enteric infections and IBD have mainly focused on their clinical and endoscopic similarities, such as severe mucosal inflammation and bloody diarrhea. In the present case, we observed a distinctive clinical presentation, namely, the coexistence of sonographic pseudo-intussusception, markedly elevated serum IgE, and concurrent *Salmonella* Typhimurium infection, which has rarely been documented together. This combination, while observed in a single case, underscores a potential diagnostic confounder that clinicians should be aware of when managing children with acute bloody diarrhea and an atopic background. Friesen et al. (9) previously reported three pediatric cases wherein acute *Salmonella* infections presented with endoscopic and clinical features closely mimicking Crohn's disease or ulcerative colitis. Similarly, a separate case report (11) noted that a multidrug-resistant (MDR) Salmonella Typhimurium infection in an elderly patient with a history of UC induced severe diarrhea and hematochezia. These atypical presentations closely resemble an acute, severe IBD flare-up. Furthermore, in the very early-onset (VEO) population, the overlap between infection and VEO-IBD often poses a significant risk of diagnostic delay. For example, Wilczyńska et al. (10) described the case of a 20-month-old infant with ulcerative colitis who initially presented with loose stools containing blood and mucus; the diagnosis was confirmed after the extensive exclusion of gastrointestinal infections and food allergies. This strongly suggests that endoscopic evaluation must be initiated as early as possible when conservative treatment proves ineffective during infancy.

**Table 1:** Review of clinical characteristics and outcomes of *Salmonella* infections complicated by inflammatory bowel disease (and mimic syndromes).

Research (Year)	Demographics (N, Age, Sex)	Pathogen/Serotype	Clinical and Endoscopic Phenotypes	Biochemical and Histopathological Findings	Treatment Strategies and Clinical Outcomes
Dronfield et al. (1974) (8)	5 adult patients (aged 18–55)	Non-typhoidal *Salmonella* (NTS): Case 1, 2: *S*. Typhimurium Case 3: *S*. Agona Case 4: Unspecified *Salmonella* Case 5: *S*. Bredeney	Clinical: Presentations consistent with ulcerative colitis (UC) Endoscopy: Severe proctitis/sigmoiditis with friable mucosa and bleeding.	Biochemistry: Patients exhibited varying degrees of anemia and inflammatory responses. Pathology: The biopsy results are consistent with UC.	Interventions and Outcomes: Case 1: Treated with oral prednisolone alone, without systemic antibiotics, and died of *Salmonella* septicemia. Case 3: Treated with glucocorticoids alone, without parenteral antibiotics, and recovered. Cases 2, 4, and 5: All recovered after receiving corticosteroid therapy (with sulphasalazine included for Case 2) in combination with parenteral ampicillin.
Friesen et al. (2008) (9)	Case 1: 5 years/Female	Case 1:*S*. Enteritidis	Clinical: Abdominal pain, diarrhea, and fever; nausea/vomiting (Cases 2, 3)	Biochemistry: Normal WBC; elevated CRP (Cases 1, 2) and ESR (Case 2).	Interventions and Outcomes: All patients received initial treatment for IBD; once the causative agent was identified, Case 1 was started on ciprofloxacin and gradually tapered off prednisone; Case 2 discontinued the previous medication and switched to intravenous ceftriaxone, followed by oral cefixime;
	Case 2: 11 years/Female	Case 2:*S*. Typhimurium	Endoscopy: Case 1: Segmental deep ulceration (CD mimic).	Pathology: Inflammatory infiltration, no granulomas (Case 2 had a crypt abscess)	Case 3 discontinued the previous medication and was started on ciprofloxacin. All patients achieved long-term remission.
	Case 3: 8 years/Male	Case 3:*S*. Typhimurium	Case 2: Pancolitis (UC mimic).		
			Case 3: Diffuse colitis with ileal nodules.		
Wilczyńska et al. (2018) (10)	20 months/Male	Negative for pathogenic microorganisms	Clinical: Severe mucoid and bloody stools, accompanied by fever and acute weight loss in the later stages of the disease;	Biochemistry: Progressive anemia (Hb as low as 8.6 g/dL);	Intravenous methylprednisolone, ceftriaxone, and metronidazole, followed by mesalazine and intravenous iron; clinical remission was achieved 8 weeks after discharge, with mesalazine alone for maintenance.
			Endoscopy: Severe continuous mucosal inflammation from the rectum to the hepatic flexure.	Pathology: Biopsy confirmed cryptitis and crypt abscesses.	
Secunda et al. (2021) (11)	69 years old/Male	Multidrug resistance (MDR) *S*. Typhimurium	Clinical: In a patient with known UC, increased bowel frequency mimicking an acute IBD flare-up.	Biochemical tests: PCR-based molecular typing confirmed isolates carrying two extended-spectrum *β*-lactamase resistance genes (*blaTEM* and *blaCTX-M*).	Mesalazine and azathioprine were continued. Based on antibiotic susceptibility testing, intravenous metronidazole and oral co-trimoxazole were added. The patient's condition improved after 4 days, and he was discharged.
			Endoscopy: Colonoscopy revealed multiple superficial ulcers	Pathology: Acute and chronic inflammatory changes.	
Yang et al. (2022) (6)	13 years old/Female	No pathogens detected (Autoimmune-mediated)	Clinical: Intestinal pseudo-obstruction, no fever, recurrent bilious vomiting.	Biochemical tests: ANA positive (1:320), PR3-ANCA significantly elevated (30x upper limit of normal); low CRP.	This manifested as corticosteroid resistance and dependence; following treatment with infliximab, PR3-ANCA titers decreased significantly, and the patient achieved clinical remission lasting more than two years.
			Endoscopy: Gastroduodenoscopy revealed superficial gastritis and duodenal dilatation; colonoscopy revealed pancolitis with diffuse ulcers.	Pathology: High *in situ* expression of PR3 in neutrophils within colonic crypt abscesses.	
Present Case	5 years/Male	*S*. Typhimurium	Clinical: Ultrasound revealed a “concentric ring sign” in the small intestine (pseudo-intussusception); CT showed significant sigmoid colon thickening.	Biochemical tests: Serum total IgE significantly elevated (peaking at 2,965 IU/mL); stool culture positive.	Systemic antimicrobial therapy (IV cefotaxime sodium) combined with retention enemas (dexamethasone and mesalazine). Symptoms resolved after 10 days, meeting discharge criteria.
			Endoscopy: Diffuse congestion, edema, and erosions, as well as scattered superficial ulcers, are present in the rectum and sigmoid colon.	Pathology: Disruption of crypt architecture; extensive infiltration of mixed inflammatory cells (including plasma cells) within the lamina propria, accompanied by cryptitis.	

ANA, antinuclear antibody; CD, Crohn’s disease; CRP, C-reactive protein; CT, computed tomography; ESR, erythrocyte sedimentation rate; g/dL, grams per deciliter; Hb, hemoglobin; IBD, inflammatory bowel disease; IgE, immunoglobulin E; IU/mL, international units per milliliter; IV, intravenous; MDR, multidrug-resistant; N, number; NTS, non-typhoidal *Salmonella*; PCR, polymerase chain reaction; PR3, proteinase 3; PR3-ANCA, proteinase 3 antineutrophil cytoplasmic antibody; S., *Salmonella*; UC, ulcerative colitis; WBC, white blood cell; *blaCTX-M*, CTX-M beta-lactamase; *blaTEM*, TEM beta-lactamase.

Another clinical feature of this case was a significant elevation in serum total IgE levels, which peaked at 2965 IU/mL. Hyper-IgE syndrome (HIES) was excluded based on the absence of hallmark recurrent staphylococcal cold abscesses and specific skeletal developmental abnormalities. Common etiologies for elevated IgE, such as parasitic infections, were carefully considered but were tentatively excluded due to the absence of Charcot-Leyden crystals on repeated stool microscopy and inconsistent clinical presentations. Given the patient's existing atopic predisposition (manifested by documented mild rashes and environmental allergies, with no clinical evidence of asthma or specific food allergies identified during the current admission), this extreme elevation in serum IgE may reflect an amplified secondary Th2-polarized immune response, potentially associated with severe impairment of the intestinal mucosal barrier. In such atopic individuals, the dual insult of bacterial invasion and chronic colonic ulceration facilitates the translocation of luminal antigens into the bloodstream, thereby inducing and amplifying a systemic Th2-polarized immune response. In very early-onset inflammatory bowel disease, approximately 10%–15% of patients may have an underlying monogenic condition; elevated IgE levels are also found in certain monogenic defects (e.g., FOXP3, IL-2RA, IKBKG, WAS, DOCK8), and a multidimensional taxonomy has been established for monogenic IBD, highlighting the importance of genetic evaluation (12).

Pediatric acute severe colitis is a medical emergency that demands a standardized proactive approach, including timely multidisciplinary interventions and strict exclusion of superimposed enteric infections prior to intensive immunosuppressive therapies (13). While intravenous corticosteroids are the guideline-recommended first-line therapy for such severe flares (13), an individualized clinical approach is imperative when formulating treatment strategies for a dual pathology comprising bacterial infection and immune-mediated inflammation; monotherapy with high-dose corticosteroids carries a profound risk of facilitating systemic pathogen dissemination. Similarly, to minimize the risk of intestinal perforation and further infection dissemination during the acute phase, limiting the initial endoscopic assessment to the rectosigmoid region and deferring a full gastrointestinal evaluation until the infectious episode resolves may be considered. Dronfield et al. (8) noted that the use of corticosteroids to treat such co-infections in the absence of effective antibiotic coverage may increase the incidence of systemic spread of pathogens and sepsis. In this case, given the patient's lack of response to outpatient oral antibiotics and the severity of the lower gastrointestinal bleeding, systemic antimicrobial therapy was administered via intravenous third-generation cephalosporins. This was combined with a retention enema containing dexamethasone, mesalazine, and Yunnan Baiyao. The inclusion of Yunnan Baiyao was intended as an empiric local adjunctive therapy to promote local hemostasis and mucosal repair, while intravenous etamsylate was concurrently utilized to support systemic hemostasis. Local rectal administration delivers the medication directly to the site of inflammation, thereby increasing local drug concentrations in the affected mucosal area while mitigating systemic corticosteroid exposure and associated systemic adverse reactions (14, 15). Furthermore, considering that both *Salmonella* infection and severe colonic inflammation can disrupt gut microbiota, the subsequent administration of a multi-strain probiotic formulation was utilized as a supportive measure to help restore intestinal microbiome homeostasis. Additionally, considering the patient's pronounced atopic background, oral cetirizine was incorporated into the supportive care regimen to manage the underlying atopic reactivity. Regarding the clinical outcome, the patient was safely discharged upon achieving complete clinical remission, which met the clinical criteria for discharge, even though the stool culture remained positive for *Salmonella*. This clinical-microbiological discordance highlights that clinical recovery often precedes pathogen clearance; therefore, instructing the family to implement rigorous household infection-control measures is a critical component of the post-discharge strategy to prevent secondary transmission. For the same reason, a short course of oral antibiotics upon discharge may be considered as consolidation therapy, and scheduling a repeat stool culture is advised to confirm *Salmonella* clearance.

Several limitations of this case report should be acknowledged. First, due to objective constraints during the acute presentation, comprehensive immunological and genomic assessments (e.g., immunoglobulins, lymphocyte subsets, and whole-exome sequencing) were not performed. Given that monogenic disorders are important considerations in VEO-IBD, genetic evaluation may be considered to help exclude primary immunodeficiencies. The absence of molecular confirmation may contribute to diagnostic uncertainty, as overlapping features between severe infectious colitis and monogenic defects may be difficult to definitively differentiate without further testing. Second, while short-term clinical stabilization was achieved, comprehensive long-term follow-up data—including endoscopic reassessment and serum IgE trends—are currently unavailable, necessitating cautious interpretation of the disease course. Further genetic and immunological testing, including targeted screening for monogenic defects associated with VEO-IBD, is planned during longitudinal follow-up, and will be initiated upon predefined clinical triggers such as disease relapse, steroid refractoriness, or failure to thrive. Future studies employing transcriptomics, proteomics, and metabolomics will facilitate better characterization of individual genetic mutations in monogenic inflammatory bowel disease, highlighting the importance of comprehensive genetic evaluation (12).

## 4 Conclusion

Pediatric patients presenting with acute lower gastrointestinal bleeding may exhibit sonographic findings mimicking mechanical obstruction, alongside concurrent bacterial infections confirmed via stool culture. Should the clinical course diverge from typical self-limiting enteritis, early endoscopic and histological evaluations are imperative to evaluate for underlying VEO-IBD. VEO-IBD complicated by an acute *Salmonella* infection presents a highly specific dual pathological condition. In such scenarios, administering a combination of local corticosteroid and 5-aminosalicylic acid (5-ASA) retention enemas under adequate systemic antimicrobial coverage appeared to be a feasible and well-tolerated approach in this specific patient. This approach may help balance the need for targeted anti-inflammatory therapy with the prevention of systemic pathogen dissemination.

## 5 Patient perspective

Initially, the patient's family expressed profound concern regarding the acute hematochezia, specifically citing an intense fear that the child would require surgical bowel resection. After the clinical team elucidated the diagnostic value of multimodal imaging—specifically CT and gastrointestinal contrast studies—the family agreed to a non-operative medical management strategy. During the intervention period, the family encountered initial adherence challenges due to the child's discomfort and anxiety during the retention enemas; however, with consistent nursing support and behavioral distraction techniques, they cooperated effectively with both intravenous medication administration and local enema care. Following the resolution of clinical symptoms, the family accepted the therapeutic outcomes and agreed to adhere strictly to the post-discharge dietary management and the long-term outpatient follow-up plan.

## Data Availability

The original contributions presented in the study are included in the article/Supplementary Material, further inquiries can be directed to the corresponding author.
